# Graves' Disease Causing Pancytopenia and Autoimmune Hemolytic Anemia at Different Time Intervals: A Case Report and a Review of the Literature

**DOI:** 10.1155/2013/194542

**Published:** 2013-11-11

**Authors:** Peyman Naji, Geetika Kumar, Shabana Dewani, William A. Diedrich, Ankur Gupta

**Affiliations:** ^1^Department of Internal Medicine, Dayton Veterans Affair Medical Center, Boonshoft School of Medicine, Wright State University, 4100 West Third Street, Dayton, OH 45428, USA; ^2^Department of Hematology and Oncology, Dayton Veterans Affair Medical Center, Boonshoft School of Medicine, Wright State University, 4100 West Third Street, Dayton, OH 45428, USA; ^3^Department of Pathology, Dayton Veterans Affair Medical Center, Boonshoft School of Medicine, Wright State University, 4100 West Third Street, Dayton, OH 45428, USA; ^4^Department of Endocrinology and Metabolism, Dayton Veterans Affair Medical Center, Boonshoft School of Medicine, Wright State University, 4100 West Third Street, Dayton, OH 45428, USA

## Abstract

Graves' disease (GD) is associated with various hematologic abnormalities but pancytopenia and autoimmune hemolytic anemia (AIHA) are reported very rarely. Herein, we report a patient with GD who had both of these rare complications at different time intervals, along with a review of the related literature. The patient was a 70-year-old man who, during a hospitalization, was also noted to have pancytopenia and elevated thyroid hormone levels. Complete hematologic workup was unremarkable and his pancytopenia was attributed to hyperthyroidism. He was started on methimazole but unfortunately did not return for followup and stopped methimazole after a few weeks. A year later, he presented with fatigue and weight loss. Labs showed hyperthyroidism and isolated anemia (hemoglobin 7 g/dL). He had positive direct Coombs test and elevated reticulocyte index. He was diagnosed with AIHA and started on glucocorticoids. GD was confirmed with elevated levels of thyroid stimulating immunoglobulins and thyroid uptake and scan. He was treated with methimazole and radioactive iodine ablation. His hemoglobin improved to 10.7 g/dL at discharge without blood transfusion. Graves' disease should be considered in the differential diagnosis of hematologic abnormalities. These abnormalities in the setting of GD generally respond well to antithyroid treatment.

## 1. Introduction

Hematologic involvement is not uncommon in Graves' disease (GD) and can have a wide spectrum. Pancytopenia and autoimmune hemolytic anemia (AIHA) are two rare complications of the GD. For the first time in the literature, we report a patient who had both of these complications at different time intervals.

## 2. Case Presentation

A 70-year-old African-American man with history of hypertension, atrial fibrillation, and congestive heart failure (CHF) presented to our hospital with increased shortness of breath and lower extremity edema. He was admitted with diagnosis of CHF exacerbation. During hospital stay, he was noted to have new-onset pancytopenia (white blood cell (WBC) 2.5 t/cmm, hemoglobin 9 g/dL, mean corpuscular volume (MCV) 89.5 fL, red cell distribution width (RDW) 15.9%, and platelets 80 t/cmm). A complete hematologic assessment including peripheral blood smear, bone marrow biopsy, and flow cytometry was unremarkable. Patient had a normocellular bone marrow with mild left myeloid shift and with adequate iron stores. Further workup revealed elevated thyroid hormone levels: serum thyroid stimulating hormone (TSH) 0.01 *μ*IU/mL and serum free-T4 (FT4) level 4.5 ng/dL (normal range for TSH 0.35–5.5 *μ*IU/mL and for FT4 0.89–1.76 ng/dL). Lab results are summarized in Table 1. CHF exacerbation and pancytopenia were attributed to hyperthyroidism and patient was started on methimazole with plans for further evaluation and treatment as outpatient. Patient's cell counts started to improve; however, he unfortunately did not present for further follow-up visits and stopped taking methimazole after a few weeks. 

One year later, he presented to hospital with fatigue, weight loss, failure to thrive, and acute kidney injury. Physical exam was notable for diffuse nontender enlargement of thyroid gland and mild tremors. Patient also had warm and moist skin with mild jaundice. There was no lymphadenopathy or splenomegaly and examination of cardiovascular and respiratory systems was unremarkable. Labs showed serum creatinine 1.4 g/dL (baseline 0.7 g/dL), TSH 0.02 uIU/mL, FT4 2.9 ng/L, WBC 7.6 t/cmm, hemoglobin 7 g/dL, MCV 94.9 fL, RDW 22.5%, and platelets 248 t/cmm. Urinalysis revealed clear urine without protein, blood, leukocytes, or casts. Additional anemia workup showed positive direct antiglobulin test (IgG, warm), low haptoglobin (<15 mg/dL), elevated reticulocyte count (10.5%), and reticulocyte index (2.37). He also had elevated bilirubin (3.6 mg/dL) and lactate dehydrogenase levels (478 IU/L). Patient had low folic acid (1.77 ng/mL, deficiency if <3.37 ng/mL) and elevated vitamin B12 (1439 pg/mL). Lab results are summarized in Table 1. Peripheral smear was notable for marked anisocytosis and some spherocytes, but no schistocytes. He had normal coagulation profile. He was diagnosed with warm AIHA and was started on glucocorticoids (prednisone 70 mg/day) and folic acid. 

Simultaneously, diagnosis of GD was confirmed by elevated levels of thyroid stimulating immunoglobulins (257% of reference control, normal <140%) and diffuse thyromegaly and increased uptake (35% at 24 hours) on radioactive iodine thyroid uptake and scan. Patient's AIHA was believed to be a complication of GD. He did not have evidence of lymphoproliferative disorder and was not on any medications known to cause AIHA. He had positive anti-nuclear antibody but had no evidence of systemic lupus erythematous. Patient did not have evidence of Graves' ophthalmopathy and underwent radioactive iodine ablation with 21.6 mCi of I-131 and was started on methimazole 10 mg/day. With medical treatment his hemoglobin improved to 10.7 g/dL at discharge without blood transfusion. Of note, he had cross-reactivity to all available blood types in the blood bank and transfusion was avoided since he did not have any symptoms of anemia, other than fatigue. Patient was treated with glucocorticoids for about a month. His lab work at 6-month followup showed hemoglobin 12.4 g/dL, TSH 7.610 uIU/mL, and FT4 0.9 ng/L on methimazole. Methimazole was stopped and the patient was started on thyroxine.

## 3. Discussion

The hematopoietic system is affected in hyperthyroidism and changes can be seen in all three blood cell lineages [1], although they are usually not catastrophic [2]. Graves' disease is the most common cause of hyperthyroidism and results from production of autoantibodies that bind to and stimulate the TSH receptors [3]. Complications of GD occur by two main mechanisms: one is the ensuing thyrotoxic state which can affect almost every organ system in the body [3] and second is the concurrent autoimmune process and association with other autoimmune diseases [3, 4].

Anemia can be seen in 10–20% of patients with thyrotoxicosis [2]. Interestingly, hyperthyroidism is associated with increased total number of red blood cells, likely due to increased tissue oxygen demands resulting in increased erythropoietin secretion. However, anemia can be seen in hyperthyroid patients because of simultaneous increase in plasma volume, shorter erythrocyte life span, abnormal iron utilization, or deficiency of iron, vitamin B12, or folate [1, 2]. In one study, anemia was present in 33% of GD patients, and an obvious secondary cause could be found only in one-third of the anemic patients [5]. The anemic patients without a clear etiology were said to have “GD Anemia.” Interestingly, their lab profile had similarities with anemia of chronic disease and their anemia improved with treatment of GD. 

Well-known autoimmune processes leading to anemia in Graves' disease are pernicious anemia, celiac disease (causing iron deficiency), and autoimmune hemolytic anemia. Pernicious anemia and celiac disease are each seen in about 1% of GD patients [4] but AIHA occurs less frequently and is limited to single case reports [6]. AIHA in the setting of GD can happen alone [7–10] or along with immune thrombocytopenic purpura, as a part of Evan's syndrome [11, 12]. The exact pathophysiology remains unclear but seems to be related to both hyperthyroidism and autoimmunity. Most of the cases [8–12], but not all [7], have been hyperthyroid at the time of diagnosis of AIHA. Being euthyroid at the time of AIHA points to the autoimmune process as the likely underlying mechanism. In addition, one can postulate that increased thyroid activity in hyperthyroid patients is also a direct result of increased autoimmunity in GD. Nevertheless, AIHA has been successfully treated in some patients solely by antithyroid medications (propylthiouracil) without glucocorticoids [10, 11], which emphasizes the role of hyperthyroidism. Regardless of pathophysiology, AIHA in the setting of GD generally responds well to treatment. Glucocorticoids are the first-line agents for treatment of idiopathic warm AIHA [13] and they seem to be effective in the setting of GD as well. We treated our patient with glucocorticoids (and methimazole) with a dramatic result and his hemoglobin increased from the nadir of 6.6 g/dL to 10.7 g/dL in a span of three weeks without any blood transfusion. 

Pancytopenia in the setting of hyperthyroidism has also been reported in a handful of cases in the literature. Most cases have been associated with Graves' disease [14–17] and one case with toxic adenoma [18]. In one case report, the patient had pancytopenia as a result of both endogenous and exogenous thyroid hormone excess at different time intervals [17]. Patient initially presented with pancytopenia and Graves' disease and was treated with radioiodine thyroid ablation leading to normal cell counts. Five years later, patient had another episode of milder pancytopenia when she was inadvertently taking excessive levothyroxine. Her cell counts normalized again when she reached euthyroid state. Fascinatingly, her bone marrow was normocellular at the first episode of pancytopenia but hypercellular at the second [17]. Based on these observations both hyperthyroidism and autoimmunity contribute to the pathogenesis of pancytopenia in GD patients. Bone marrow biopsy can be hypercellular [14, 15] or normocellular [17, 19] (like our patient) and theories such as organ sequestration, decreased peripheral circulation times, stem cell dysfunction, and nutritional deficiencies have been suggested.

One potential concern in management of patients with GD and pancytopenia can be the use of antithyroid medications. Thionamides are the commonly prescribed anti-thyroid drugs with known adverse effect of agranulocytosis and aplastic anemia [20]. Using these in a patient with pancytopenia can be challenging. However, the published cases in the literature have almost universally responded well to treatment with these medications, and reaching euthyroid status has been the key for correction of the hematologic abnormality [14–19]. This has also been true in cases of patients with autoimmune hemolytic anemia and Evan's syndrome [8–12]. Therefore, hematologic complications of GD are not a contraindication to thionamides and could be an indication for prompt use in certain situations as mentioned above. 

It is not clear why our patient transformed from pancytopenia to AIHA within a year. One patient in the literature had transformed from pancytopenia to aplastic anemia (on concomitant methimazole) [15], and another patient with recurrent AIHA has had a transient pancytopenia simultaneous with one of his AIHA episodes [8]. Our patient is the first case who had pancytopenia followed by AIHA. He had untreated Graves' disease for most of his clinical course which possibly could be responsible for this transformation. 

## 4. Conclusion

Graves' disease can cause various changes in hematopoietic system and should be considered in the differential diagnosis of hematologic abnormalities. These abnormalities in the setting of GD generally respond well to anti-thyroid treatment and reaching euthyroid status should be set as the treatment goal in these patients. 

## Figures and Tables

**Table 1:** Summary of lab results during the first and second hospital admissions.

Lab test	Normal range	First admission (pancytopenia)	Second admission (AIHA)
White blood cells	4.8–10.8 t/cmm	2.5	7.6
Hemoglobin	14–18 g/dL	9	7
MCV	80–94 fL	89.5	94.9
RDW	11.5–14.5%	15.9	22.5
Platelets	130–400 t/cmm	80	248
Reticulocyte count	0.5–1.5%	1	10.5
LDH	98–192 IU/L	193	478
Total bilirubin	0.2–1.5 mg/dL	2	3.6
Direct Coombs test	Negative	Negative	Positive (IgG)
TSH	0.35–5.5 *μ*IU/mL	0.01	0.02
Free T4	0.89–1.76 ng/dL	4.5	2.9

AIHA: autoimmune hemolytic anemia, MCV: mean corpuscular volume, RDW: red cell distribution width, LDH: lactate dehydrogenase, and TSH: thyroid stimulating hormone.
